# Comparison of Three Computational Tools for the Prediction of RNA Tertiary Structures

**DOI:** 10.3390/ncrna10060055

**Published:** 2024-11-08

**Authors:** Frank Yiyang Mao, Mei-Juan Tu, Gavin McAllister Traber, Ai-Ming Yu

**Affiliations:** Department of Biochemistry and Molecular Medicine, School of Medicine, University of California Davis, 2700 Stockton Blvd, Sacramento, CA 95817, USA

**Keywords:** RNA structure, 3D structure, computational modeling, tRNA, therapeutic RNA, RNA interference, microRNA, recombinant RNA

## Abstract

Understanding the structures of noncoding RNAs (ncRNAs) is important for the development of RNA-based therapeutics. There are inherent challenges in employing current experimental techniques to determine the tertiary (3D) structures of RNAs with high complexity and flexibility in folding, which makes computational methods indispensable. In this study, we compared the utilities of three advanced computational tools, namely RNAComposer, Rosetta FARFAR2, and the latest AlphaFold 3, to predict the 3D structures of various forms of RNAs, including the small interfering RNA drug, nedosiran, and the novel bioengineered RNA (BioRNA) molecule showing therapeutic potential. Our results showed that, while RNAComposer offered a malachite green aptamer 3D structure closer to its crystal structure, the performances of RNAComposer and Rosetta FARFAR2 largely depend upon the secondary structures inputted, and Rosetta FARFAR2 predictions might not even recapitulate the typical, inverted “L” shape tRNA 3D structure. Overall, AlphaFold 3, integrating molecular dynamics principles into its deep learning framework, directly predicted RNA 3D structures from RNA primary sequence inputs, even accepting several common post-transcriptional modifications, which closely aligned with the experimentally determined structures. However, there were significant discrepancies among three computational tools in predicting the distal loop of human pre-microRNA and larger BioRNA (tRNA fused pre-miRNA) molecules whose 3D structures have not been characterized experimentally. While computational predictions show considerable promise, their notable strengths and limitations emphasize the needs for experimental validation of predictions besides characterization of more RNA 3D structures.

## 1. Introduction

Noncoding RNAs (ncRNAs), such as microRNAs (miRNAs) and transfer RNAs (tRNAs), play important roles in the initiation, progression, and therapeutic resistance of various diseases, which are often associated with poor prognosis and limited treatment options [1,2]. Recent advancements in developing RNA-based therapeutics, including RNA drugs and RNA-targeted small-molecule drugs, underscore the potential of targeting RNAs involved in key pathways underlying disease progression [2,3,4,5,6]. Among them, RNA interference (RNAi) molecules, such as small interfering RNAs (siRNAs) or miRNA mimics or biosimilars [2,3,7], have garnered considerable attention due to their versatile and robust gene regulation capabilities. In particular, the antisense or guide strands from the siRNA or miRNA duplexes are incorporated into the RNA-induced silencing complex (RISC), where they act on specific mRNAs for translational inhibition and degradation to achieve disease control [2,3,7]. On the other hand, small molecules may be employed to directly target primary or precursor miRNA (pri- or pre-miR) species within the RNAi machinery for the management of relevant diseases [8,9].

Knowledge of RNA tertiary (3D) structures should improve the understanding of structure–function relationships, interactions with respective RNA binding proteins, and the development of RNA-based therapeutics, such as those reliant on RNAi processes. Indeed, many X-ray crystallography and cryo-electron microscopy (cryo–EM) studies [10,11,12,13,14,15] have been conducted to characterize the structures of Dicer in the absence and presence of RNAs, as well as variable conformations of the RISC complexes, providing structural insights into RNA-protein interactions and RNAi. This growing body of structural knowledge is expected to continue facilitating the design and development of new RNAi therapeutics and small molecule modulators [8,9,16].

However, RNA molecules inherently exhibit less pronounced hydrophobic and electrostatic interactions compared to proteins, leading to a greater diversity of 3D structures influenced by factors beyond just their primary and secondary structures [17]. This high flexibility and diverse folding potential pose significant challenges for modern experimental technologies, including X-ray crystallography and cryo–EM [18,19]. The RNAs may resist the uniform crystallization required for X-ray crystallography, even with the aid of binding proteins or stabilizing agents. Similarly, the lack of uniform folding and low homogeneity in RNAs make it difficult to obtain high-resolution structures via cryo–EM unless computational interpretations are employed to generalize structural characterization [18,20]. Hybrid methods, such as small-angle X-ray scattering (SAXS) combined with all-atom molecular dynamics (MDs) simulations, also face challenges in resolving single high-resolution RNA 3D structures [19,21].

Consequently, despite the identification of thousands of ncRNA species, only a limited number of RNA 3D structures have been experimentally determined, most of which are RNA-protein complexes [18,20,22]. This limitation emphasizes the indispensable role of computational approach. Coupled with advancements in de novo RNA 3D structure prediction programs like AlphaFold 3 [23] and Rosetta Fragment Assembly of RNA with Full-Atom Refinement 2 (FARFAR2) [24], computational methods are becoming increasingly valuable for gaining structural insights [25]. Current computational tools, including coarse-grained (CG) MD simulations (e.g., VFold) [26,27], motif assembly (e.g., RNAComposer) [28], fragment assembly (e.g., FARFAR2) [23], and deep-learning platforms (e.g., AlphaFold 3) [23], offer promising methods for constructing 3D structures based on databases of experimentally determined RNA structures. While VFold has limitations in predicting diverse structural folding [26,27], RNAComposer, FARFAR2, and AlphaFold 3 have garnered significant recognition in the RNA research community. These programs provide web server access, facilitating de novo RNA 3D structure prediction. However, despite the well-documented architecture and performance trials of each program in their respective publications, their applicability and limitations in predicting RNA 3D structures remain unclear. This ambiguity can make selecting the most appropriate computational tool for specific research objectives challenging, especially without a deep understanding of computational linguistics and structural biology.

In this study, we employed RNAComposer, FARFAR2, and AlphaFold 3 to predict the 3D structures of various forms of RNAs, including one representative siRNA drug [2,4,7] and those relevant to our novel bioengineered RNA (BioRNA) molecules with therapeutic potential [29,30,31,32,33,34,35,36]. Differences among the computationally predicted 3D structures versus the experimentally determined counterparts were compared, and their strengths and limitations were noted. Our results showed that, overall, AlphaFold 3 demonstrated greater accuracy than RNAComposer and FARFAR2 in the prediction of RNA structures with 3D structures that have been determined experimentally. Nevertheless, significant variations persisted across different computational tools when predicting RNAs with unknown 3D structures, underscoring the continued need for experimental validation, including larger RNA molecules such as recombinant BioRNAs.

## 2. Results

### 2.1. Prediction and Comparison of the Malachite Green Aptamer (MGA) 3D Structures

**Figure 1:**
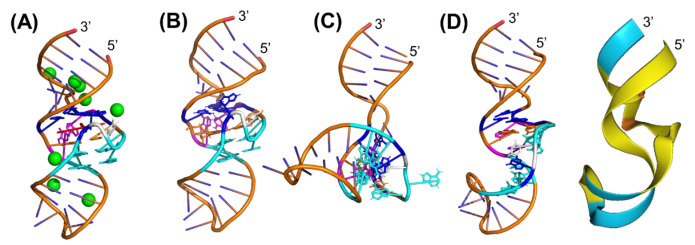
Malachite green aptamer (MGA) 3D structures predicted by different computational programs and compared with the crystal structure. (**A**) The 2.8 Å crystal structure of MGA with strontium ions (green) and tetramethylrosamine (TMR, red) (PDB ID: 1f1t). Segments are color coded as follows: base quadruple (G24∙A31∙G29:C7, blue) above TMR, two sets of base triples (A26∙U11:A22 and A27∙C10:G23, cyan) below the TMR, pair of stacking bases (magenta) adjacent to TMR, and U-turn bulge (U25, white). (**B**) The MGA 3D structure predicted by RNAComposer. Compared with the crystal structure (**A**) by using PyMOL 3.0, it shows the all-atom root mean square deviation (RMSD) difference of 2.558 Å. (**C**) The MGA 3D structure predicted by Rosetta FARFAR2 exhibits an RMSD value of 9.702 Å relative to the crystal structure (**A**). (**D**) The MGA 3D structure predicted by AlphaFold 3, with an RMSD of 5.745 Å relative to the crystal structure (**A**). Prediction confidence is illustrated by color in ribbon form as follows: blue for high confidence regions, yellow for low confidence regions, and orange for very low confidence regions.

We first chose a relatively shorter RNA for the comparative study, MGA (38 nt in length), with a 3D crystal structure that has been resolved to a 2.8 Å resolution in conjunction with tetramethylrosamine (TMR) (Figure 1A) [37]. It consists of extensive 5-bromouridine chemical modification, which was thought to have no effect on MGA tertiary folding [37]. After obtaining its primary sequence from the Protein Data Bank (PDB) (PDB ID: 1f1t), we predicted its secondary structure with RNAfold and CONTRAfold, which were identical (Table S1 and Figure S1) and served as the input for the RNA 3D structure predictions [38] by RNAComposer and Rosetta FARFAR2.

Our results showed that the MGA 3D structure predicted by RNAComposer (Figure 1B) successfully recapitulated its crystal structure, including all base pairing and stacking interactions, which has a high degree of structural similarity also indicated by a low all-atom root mean square deviation (RMSD) (2.558 Å) after superimposition and analysis in PyMOL 3.0. By contrast, the 3D structure predicted by Rosetta FARFAR2 (Figure 1C) showed significant over-twisting of the hairpin loop and lacked the coaxial base stacking observed in the crystal structure, giving a higher RMSD of 6.895 Å that exceeds the RMSD_100_ threshold of 4.0 Å for small RNA structures [24]. Furthermore, AlphaFold 3 produced a 3D structure (Figure 1D) directly from the input primary sequence that approximated the crystal structure (RMSD of 5.745 Å). However, it was associated with a lower prediction confidence in the overall conformation.

### 2.2. Structural Prediction and Validation of Human Glycyl-tRNA-CCC

**Figure 2:**
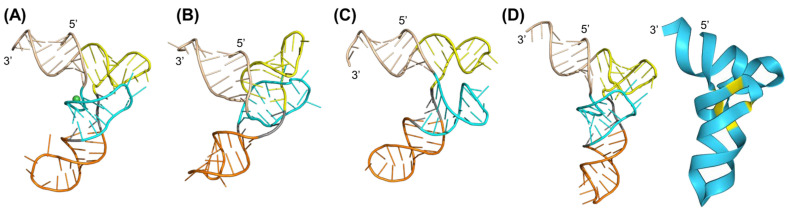
Comparison of human glycyl-tRNA-CCC (htRNA^Gly-CCC^) 3D structures determined experimentally (**A**) and predicted by RNAComposer (**B**), Rosetta FARFAR2 (**C**), and AlphaFold 3 (**D**). (**A**) The htRNA^Gly-CCC^ 3D structure is extracted from the human glycyl-tRNA synthetase complex (hGlyRS–htRNA^Gly-CCC^) crystal structure (PDB ID: 5E6M, 2.93 Å resolution) and annotated according to its secondary structure prediction from RNAfold as follows: acceptor arm (wheat tint), D arm (cyan), T arm (yellow), and anticodon arm (orange). (**B**) The htRNA^Gly-CCC^ 3D structure predicted by RNAComposer shows an RMSD of 16.077 Å as compared to the crystal structure (**A**). (**C**) The htRNA^Gly-CCC^ 3D structure predicted by Rosetta FARFAR2, with an RMSD of 7.482 Å relative to the crystal structure (**A**). (**D**) The htRNA^Gly-CCC^ 3D structure predicted by AlphaFold 3 exhibits an RMSD of 5.522 Å relative to the crystal structure (**A**) The degree of prediction confidence is displayed in ribbon form as follows: blue is relatively high, while yellow is relatively low.

We further evaluated the utilities of the three computational programs for the prediction of 3D structures of human glycyl-tRNA-CCC (htRNA^Gly-CCC^) and compared to its crystal structure (Figure 2A) being resolved in complex with human glycyl-tRNA synthetase (PDB ID: 3a3a) at a resolution of 2.95 Å [39]. Likewise, we retrieved the htRNA^Gly-CCC^ primary sequence from the PDB and found that its secondary structure predicted by RNAfold and CONTRAfold differed slightly in the base pairings of the D arm (Table S1 and Figure S1).

When the secondary structure predicted by RNAfold was used as input, the htRNA^Gly-CCC^ 3D structure predicted by RNAComposer (Figure 2B) showed notable divergence from its crystal structure. In particular, the four-way junction was more stacked and enclosed, which reduced the loop-kissing interactions between the D and T loops. This structural alteration correlated with an over-twisting of the D stem, leading to an overall RMSD of 16.077 Å (Figure 2B), exceeding the RMSD_100_ threshold of 9.1 Å for relatively larger RNA structures [24]. By contrast, the htRNA^Gly-CCC^ 3D structure predicted by Rosetta FARFAR2 (Figure 2C), with a slightly smaller RMSD value of 7.482 Å relevant to its crystal structure, more closely replicated the overall folding. However, the D arm adopted a sharp hairpin turn that lacked a clear interaction between the D and T loops (Figure 2C). Interestingly, when the secondary structure predicted by CONTRAfold (Table S1 and Figure S1) was used as the input, the 3D structures of htRNAGly-CCC predicted by RNAComposer (Figure S2B) were much closer to its crystal structure than Rosetta FARFAR2 (Figure S2C), as manifested by the RMSD of 5.899 Å and 12.734 Å, respectively. Further, Rosetta FAFAR2 could not recapitulate the inverted “L” shape of a typical tRNA structure, whereas RNAComposer did. These results indicate the dependence on secondary structure input by RNAComposer and Rosetta FARFAR2 in their 3D structure predictions.

On the other hand, AlphaFold 3 generated a 3D structure directly from the primary sequence input with high prediction confidence (Figure 2D), displaying only a small deviation from the crystal structure, as manifested by the RMSD value of 5.522 Å that is below the RMSD_100_ threshold [24]. In addition, the AlphaFold 3 predicted structure (Figure 2D) fully characterized the D/T-loop interactions and the pocket bulge caused by the nickel ion (green sphere) near the acceptor-D stem linkage as identified by the X-ray crystallography study (Figure 2A) [39].

### 2.3. Comparison of 3D Structures of Human Isoacceptor Glycyl-tRNA-GCC Predicted by Three Computational Programs

**Figure 3:**
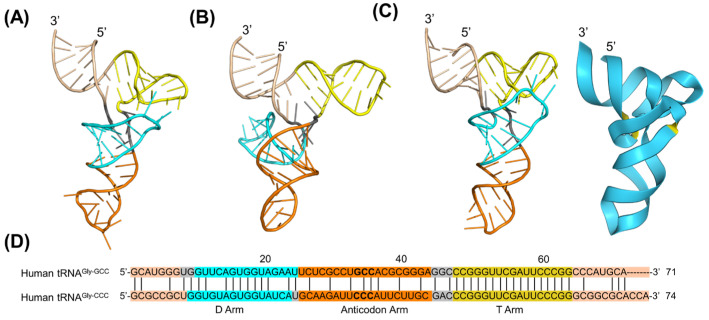
Comparison of human glycyl-tRNA-GCC (htRNA^Gly-GCC^) 3D structures predicted computationally with RNAComposer (**A**), Rosetta FARFAR2 (**B**), and AlphaFold 3 (**C**). Acceptor arm (wheat tint), D arm (cyan), T arm (yellow), and anticodon arm (orange). The blue and yellow areas in the ribbon-form structure predicted by AlphaFold 3 indicate high and low confidence in its prediction, respectively. (**D**) Comparison of the primary sequences of the htRNA^Gly-GCC^ examined herein and htRNA^Gly-CCC^ isoacceptor shown in Figure 2. Note that the secondary structure predicted by CONTRAfold was used as inputs for RNAComposer and Rosetta FARFAR2, whereas its primary sequence was directly inputted for AlphaFold 3 prediction. The secondary structure predicted by RNAfold failed to offer unpaired anticodon GCC (Figure S1), thus a common inverted “L” shape tRNA 3D structure (Figure S2).

We employed the three computational tools to predict and compare the 3D structures of human Glycyl-tRNA-GCC-1-1 isoacceptor (htRNA^Gly-GCC^) that have not been determined experimentally; it is one of the most abundant htRNAs and is being identified and used to offer a robust BioRNA technology [29,40]. Therefore, we extracted the primary sequence from tRNA database GtRNAdb and obtained its secondary structure predictions (Table S1 and Figure S1) with RNAfold and CONTRAfold. Surprisingly, the anticodon GCC was found in the hairpin stem secondary structure predicted by RNAfold, while the GCC anticodon loop was obvious within the secondary structure predicted by CONTRAfold (Figure S1). As a result, its 3D structures predicted by both RNAComposer and Rosetta FARFAR2 from the RNAfold secondary structure (Figure S3) failed to recapitulate the typical inverted “L” shape tRNA structure. Interestingly, when the CONTRAfold predicted secondary structure consisting of typical tRNA base pairing was used as input, RNAComposer successfully offered the inverted “L” shape tRNA 3D structure (Figure 3A), whereas Rosetta FARFAR 2 produced a rater “T” shape 3D structure (Figure 3B). By contrast, AlphaFold 3 readily predicted the inverted “L” shape tRNA 3D structure (Figure 3C) directly from the primary sequence input.

To further evaluate the predicted structures, the htRNA^Gly-CCC^ crystal structure (Figure 2A) was used as a reference because the two htRNA^Gly^ sequences are highly conserved (Figure 3D). The results showed that the htRNA^Gly-GCC^ 3D structure predicted by RNAComposer (Figure 3A) exhibited a smaller degree of deviation from the htRNA^Gly-CCC^ crystal structure, with an RMSD of 5.765 Å. In comparison, the htRNA^Gly-GCC^ 3D structure predicted by Rosetta FARFAR2 (Figure 3B) displayed much greater deviation, with an RMSD of 15.772 Å as compared with the htRNA^Gly-CCC^ crystal structure. Notably, the D and T arms are oriented in opposing directions, with the D arm adopting a 180-degree reverse turn pointing into the page (Figure 3B). Interestingly, AlphaFold 3 also produced a htRNA^Gly-GCC^ 3D structure (Figure 3C) matching the htRNA^Gly-CCC^ crystal structure with preserved tRNA structural elements (Figure 2A), as indicated by an RMSD of 4.019 Å, which is likely related to its deep learning framework from experimentally determined 3D structures of similar RNA molecules.

### 2.4. Computational Prediction and Comparison of Human Pre-miRNA Let-7a 3D Structures with a Cryo–EM Structure

**Figure 4:**
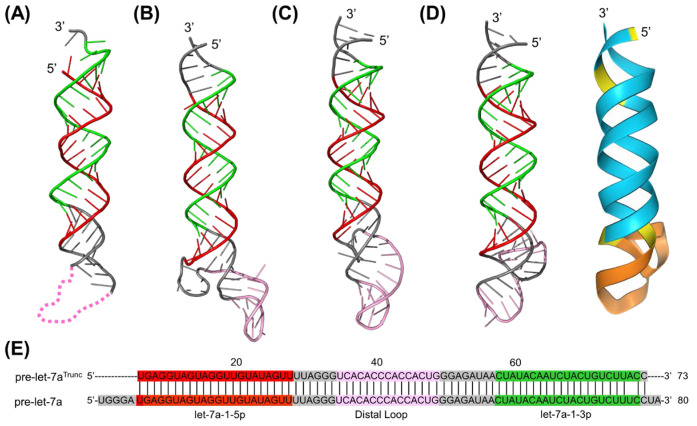
Comparison of human precursor microRNA let-7a-1 (pre-let-7a) 3D structures predicted by RNAComposer (**B**), Rosetta FARFAR2 (**C**), and AlphaFold 3 (**D**) vs. the 3D structure (**A**) of its truncated version (pre-let-7a^Trunc^) determined experimentally. (**A**) The pre-let-7a^Trunc^ 3D structure extracted from the human Dicer–TRBP complex determined by cryo–EM (PDB ID: 5ZAL, 3.1 Å resolution). Let-7a-1-5p strand (red), let-7a-1-3p (green), and distal loop (pink; orange in ribbon form), which has an undefined structure. Note that the 3D structure of the distal loop remains uncharacterized. (**B**) The pre-let-7a 3D structure predicted by RNAComposer, with an RMSD of 5.251 Å as compared to the cryo–EM structure (**A**). (**C**) The pre-let-7a 3D structure predicted by Rosetta FARFAR2 shows an RMSD of 6.037 Å relative to the crystal structure (**A**). (**D**) The pre-let-7a 3D structure predicted by AlphaFold 3, with an RMSD of 4.890 Å difference from the crystal structure (**A**). Prediction confidence is displayed in ribbon form as follows: blue relatively high, yellow relatively low, and orange very low. (**E**) Comparison of the primary sequences of the pre-let-7a^Trunc^ and pre-let-7a. The secondary structure predicted by CONTRAfold was used as the input for RNAComposer and Rosetta FARFAR2 predictions, and its primary sequence was directly inputted for AlphaFold 3 prediction.

We further investigated human pre-miRNA 3D structural predictions since it is the payload of therapeutic BioRNA agents [2,3,29]; the 3D structure of a truncated pre-let-7a (pre-let-7a^Trunc^) has been characterized in complex with human Dicer–TRBP at a resolution of 4.70 Å (PDB ID: 5ZAL) using cryo–EM technology, although the distal loop residues (U29 to G43, pink) remain unresolved due to unassigned density (Figure 4A) [12]. We retrieved the primary sequences of wild-type human pre-let-7a from miRBase [41] for 3D structure prediction by AlphaFold 3, and its secondary structures predicted by RNAfold and CONTRAfold, which exhibited an overall typical pre-miRNA stem-loop hairpin structure, but differed sharply in the terminal segment (Table S1 and Figure S1), were used for 3D structure predictions by RNAComposer and Rosetta FARFAR2 (Figure 4B–D; Figure S4). Sequence alignment (Figure 4E) illustrated the identity between pre-let-7a^Trunc^ (served as a reference) and pre-let-7a, besides a few terminal residues removed from the truncated form.

All predicted 3D structures consistently agreed on the A-form helix conformation of the let-7a duplex and matched the cryo–EM structure (Figure 4A–D; Figure S4). However, the pre-let-7a 3D structure generated by RNAComposer from the secondary structure predicted by CONTRAfold (Table S1 and Figure S1) consisted of an internal loop (Figure S4B), consistent with the secondary structure input. Nevertheless, the pre-let-7a 3D structures predicted by both RNAComposer and Rosetta FARFAR2 from the CONTRAfold secondary structure exhibited a sharp turn to offer an “L” sharp structure (Figure S4B,C), largely distinct from the truncated pre-let-7a cryo–EM structure (RMSD of 10.285 Å and 10.015 Å, respectively).

On the other hand, pre-let-7a 3D structures (Figure 4B–D) predicted from the RNAfold secondary structure (Table S1 and Figure S1) by RNAComposer and Rosetta FARFAR2 as well as primary sequence by AlphaFold 3 showed a relatively higher degree of similarity. When superimposing the cryo–EM structure onto computationally predicted 3D structures, the RMSD values were 5.251 Å for RNAComposer, 6.037 Å for Rosetta FARFAR2, and 4.890 Å for AlphaFold 3. Nevertheless, for the distal loop residues with an unresolved 3D structure (Figure 4A), all three computational tools positioned the hairpin loop in strikingly different orientations with distinct base pairings (Figure 4C,D). Indeed, very low prediction confidence is also indicated by the orange coloring for this segment in the ribbon structure generated by AlphaFold 3 (Figure 4D), suggesting a greater extent of uncertainty in the absence of experimental structures of relevant RNAs.

### 2.5. De Novo Prediction of htRNA^Gly-GCC^-Fused Human Pre-microRNA Let-7a 3D Structure

**Figure 5:**
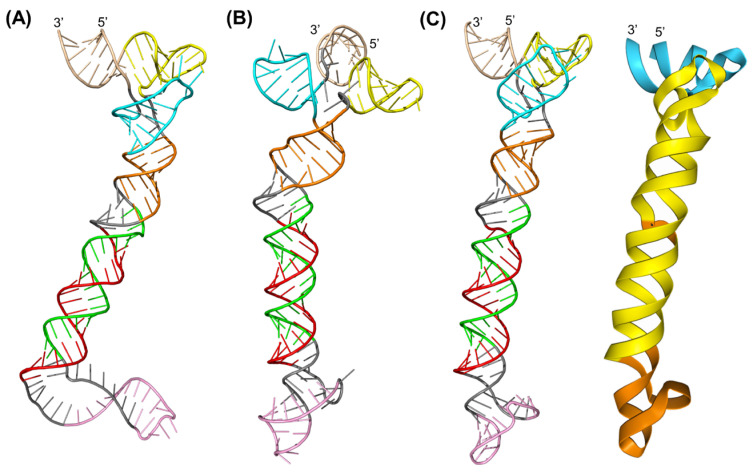
Comparison of the 3D structures of human tRNA^Gly-GCC^-fused hsa-pre-let-7a (BioRNA^Gly^/pre-let-7a) predicted computationally by RNAComposer (**A**), Rosetta FARFAR2 (**B**), and AlphaFold 3 (**C**). The tRNA segment is displayed as follows: acceptor arm (wheat tint), D arm (cyan), T arm (yellow); the pre-let-7a segment is displayed as follows: let-7a-1-5p (red), let-7a-1-3p (green), distal loop (pink; orange in ribbon form). The blue and yellow areas in the ribbon-form structure predicted by AlphaFold 3 indicate high and low confidence, respectively, while the orange denotes very low confidence in its prediction. The secondary structure predicted by CONTRAfold was used as the input for RNAComposer and Rosetta FARFAR2 predictions, and its primary sequence was directly inputted for AlphaFold 3 prediction. Note that the 3D structure of the distal loop within pre-let-7a (Figure 4A) was undefined in the cryo–EM study [12].

In addition, we employed the three computational programs to predict and compare 3D structures of one model therapeutic BioRNA [2,3,29], in particular, the htRNA^Gly-GCC^-fused human pre-miR let-7a, 148 nucleotides in length (Table S1). As expected, CONTRAfold provided a common hairpin stem loop anticodon arm as well as D arm for the htRNA^Gly-GCC^ segment (Table S1 and Figure S1), whereas RNAfold did not. By contrast, the terminal segment within the pre-let-7a was well structured by RNAfold while showing a larger internal loop by CONTRAfold. Consequently, the BioRNA 3D structures predicted by RNAComposer and Rosetta FARFAR2 from RNAfold-derived secondary structure did not show an inverted “L” shape tRNA structure (Figure S5). By contrast, the BioRNA^Gly^/pre-let-7a 3D structure predicted from CONTRAfold-generated secondary structure by RNAComposer (Figure 5A) as well as from its primary sequence by AlphaFold 3 (Figure 5C) consisted of obvious tRNA folding pattern for the htRNA^Gly-GCC^ segment, consistent with results obtained for anticodon-containing htRNA^Gly-GCC^ (Figure 3A,C). By contrast, the 3D structure generated by Rosetta FARFAR2 from the same CONTRAfold secondary structure input displayed rather dispersed structural conformations for the htRNA^Gly-GCC^ segment, lacking an inverted “L” shape structure or long-range interactions as possible constraints (Figure 5B).

The pre-let-7a segment within all three predicted 3D structures consistently displayed the A-form helix (Figure 5A–C), as observed in earlier predictions for pre-let-7a alone (Figure 4B–D). However, compared to the predicted pre-let-7a 3D structures in Figure 4, there were noticeable variations in the local conformation and orientation of the distal loop. In particular, the RNAComposer predicted 3D structure now features a large internal loop besides the original distal loop (Figure 5A), which can be traced back to the secondary structure predicted by CONTRAfold (Figure S1E). While CONTRAfold offered the typical tRNA secondary structure, it failed to include four key base pairings (G65∙U86; U66∙A85; C67∙G84; C73∙G80) in the distal hairpin stem, unlike the RNAfold secondary structure prediction (Figure S1 and Table S1). The first three consecutive base pairs divided the large internal loop into smaller internal and bulge loops that served as a structural constraint, resulting in a more compact coaxial 3D structure, as demonstrated in the earlier RNAComposer predicted structure (Figure 4C). Although a similar internal loop was present in the 3D structures predicted by Rosetta FARFAR2 and AlphaFold 3, the latter contained fewer unpaired residues that offered more compact structures. Rather, the prediction confidence for the pre-let-7a component within BioRNA (Figure 5C) by AlphaFold 3 was lower than the pre-let-7a itself (Figure 4D), underscoring the complexities involved in the structural conformation of larger RNA molecules without access to experimental structures.

### 2.6. Prediction of RNA 3D Structures Consisting of Modified Nucleotides

Lastly, we explored potential differences in the predicted RNA 3D structures with and without modified nucleotides, considering the presence of post-transcriptional modifications in natural RNAs and extensive chemical modifications applied to therapeutic RNAs [2,4,7]. Currently, RNAComposer and Rosetta FARFAR2 cannot model double-stranded RNAs or incorporate modified nucleotides. By contrast, AlphaFold 3 can model multiple RNA entities and supports a limited number of natural modifications, such as pseudouridine (Ψ), 5-methyluridine (m5U), and 2′-*O*-methylguanosine (Gm). For this analysis, we selected one siRNA drug approved most recently by the United States Food and Drug Administration, nedosiran [7], and a human leucyl tRNA isoacceptor, htRNA^Leu-UAA^, which are composed of extensive chemical modifications and several well-conserved post-transcriptional modifications (Table S2), to examine the potential effects of modified nucleotides on predicted RNA 3D structures.

**Figure 6:**
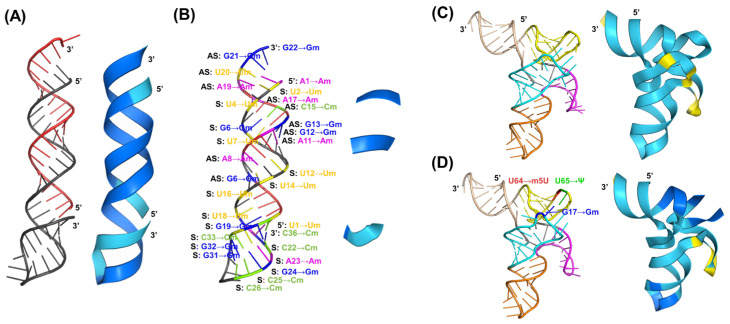
Predicted 3D structures of the siRNA drug nedosiran (**A**,**B**) and human leucyl-tRNA (htRNA^Leu-UAA^) (**C**,**D**) by AlphaFold 3. Sense (S, black) and antisense (AS, red) RNA strands are noted for the nedosiran, and the acceptor arm (wheat tint), D arm (cyan), anticodon arm (orange), variable arm (magenta), and T arm (yellow) are color coded for htRNA^Leu-UAA^. Panels **A** and **C** show unmodified RNAs, while panels **B** and **D** are respective RNAs with a few modifications supported by AlphaFold 3, including 2′-*O*-methylcytidine (Cm), 2′-*O*-methylguanosine (Gm), 2′-*O*-methyladenosine (Am), and 2′-*O*-methyluridine (Um), 5-methyluridine (m5U), and pseudouridine (ψ). See Table S2 for complete chemical modifications applied to nedosiran and conserved post-transcription modifications for htRNA^Leu-UAA^; many are currently not supported by AlphaFold 3. The blue and cyan regions in the ribbon structure represent very high and high prediction confidence, respectively, while yellow indicates low confidence. While overall 3D structures looked similar, a RMSD of 1.993 Å between unmodified and modified nedosiran siRNA (**A**,**B**) was observed, as well as 1.431 Å between modified and unmodified htRNA^Leu-UAA^. Interestingly, the inclusion of chemical modifications affected AlphaFold 3′s ability to perform local prediction confidence analysis and generate the complete ribbon structure for nedosiran siRNA (**B**), while incorporation of a few conserved post-transcriptional modifications improved the prediction confidence for surrounding regions (**D**).

Nedosiran double-stranded siRNA 3D structures predicted by AlphaFold 3, in the absence and presence of several modified nucleotides, seemed the same (Figure 6A,B), with a small RMSD value of 1.993 Å. Interestingly, the methylations of C36 and U1 led to a predicted structure in which the 3′ end of the sense strand and 5′ end of the antisense strand are bridged (Figure 6B). Likewise, inclusion of three post-transcriptionally modified nucleotides in htRNA^Leu-UAA^, G17→Gm, U64→m5U, and U65→ψ, caused a minor “pinch” in the local structure, with an RMSD of 1.431 Å when compared with unmodified version (Figure 6D). It is noteworthy that extensive chemical modifications dampened the performance of AlphaFold 3 to offer an incomplete ribbon structure for nedosiran siRNA to illustrate prediction confidence (Figure 6B). On the other hand, the application of three conserved, post-transcriptional modifications to htRNA^Leu-UAA^ obviously improved the prediction confidence for surrounding regions (Figure 6D). These results not only demonstrate the capacity of the most recent tool, AlphaFold 3, for the prediction of RNA 3D structures, but also indicate structural changes with the occurrence of nucleotide modifications; although these changes are not always visually apparent, are well-known to alter RNA metabolic stability and final bioactivity.

## 3. Discussion

Given the advancements in computational predictions of RNA structures, we employed multiple RNAs, about 40–150 nt in length, single- and double-stranded, to evaluate the utilities of three common tools, namely RNAComposer and Rosetta FARFAR2, as well as the latest AlphaFold 3, in this study. Our studies revealed apparent and variable degrees of discrepancies in the accuracy and convergence of 3D structures generated by different computational programs. The divergences become more striking for longer RNAs and any RNAs or segments with 3D structures that have not been experimentally characterized. These discrepancies are likely attributable to distinct algorithms and workflows of individual tools. In addition, the performances of RNAComposer and Rosetta FARFAR2 are highly dependent on the inputted RNA secondary structures which may be distinctly predicted by relevant tools, such as RNAfold and CONTRAfold examined herein in which RNAfold sometimes fails to offer base pairings for an inverted “L” shape tRNA structure. By contrast, AlphaFold 3 utilizes RNA primary sequences as inputs and accepts some common post-transcriptional modifications to directly generate their 3D structures, which also shows a better recapitulation of those known RNA 3D structures while indicating much lower confidence in the prediction of unknown RNA structures. Our findings on the strengths and limitations of current computational tools for the prediction of RNA 3D structures iterate the needs for more experimental RNA structures for deep learning and development of other computational or artificial intelligence (AI) methods to improve prediction accuracy, and experimental validation of computational outcomes.

The RNAComposer is featured by its tertiary structure translation workflow that divides the secondary structure into large motifs at canonical base pairs, translating each motif into 3D fragments using its RNA FRABASE database [28]. As a result, RNAComposer demonstrated exceptional accuracy in replicating the detailed folding and base pairing network of the crystal structure of MGA, a small RNA molecule of 38 nt. The RNAComposer probably treated the small MGA structure as a single motif. Another key feature of RNAComposer is its ability to modify the primary sequence input during translation if a matching secondary structure with a different sequence is identified, which might ensure the preservation of tertiary folding [28]. However, this feature likely accounts for its relatively poor performance to predict htRNA^Gly-CCC^ 3D structure, where the RNAfold-predicted secondary structure input is inconsistent with its crystal structure. The influence of secondary structure inputs on RNAComposer predictions is more obviously demonstrated in the prediction of htRNA^Gly-CCC^ 3D structures. This reliance may also explain the large internal loop in its predicted BioRNA 3D structure, where the CONTRAfold secondary structure prediction seems directly implemented without further refinement. One could potentially mitigate this limitation by using RNAComposer under batch mode with multiple inputs, not restricting it to statistical machine-learning tools like CONTRAfold for secondary structure prediction [42]. Overall, reliable secondary structure inputs can lead to appreciable performance by RNAComposer, as compared to the predictions directly from primary sequence input by AlphaFold 3.

Conversely, Rosetta FARFAR2 underperforms RNAComposer to predict MGA 3D structure, and depending upon the secondary structure inputs, it might surpass or fall behind RNAComposer in predicting 3D structures of tRNAs or larger RNAs such as therapeutic BioRNAs [3,43]. Rosetta FARFAR2 offers a more flexible approach by accepting not only primary sequence and secondary structure inputs, but also in various constraint settings, template structure files, and experimental data such as RNA chemical shifts. Its primary workflow involves segmenting the input into three residue fragments and matching each within its fragment library. This process is guided by an algorithm designed to preserve Watson–Crick base pairs and helical conformations within its database. Due to the small fragment size, Rosetta FARFAR2 employs a Monte Carlo process to assemble and refine a large pool of RNA 3D structures, generating between 3000 and 30,000 structures for each RNA and clustering the top 1% of the lowest-energy structures into more representative models [24]. In our trials using the Rosetta online server, we observed that the lowest energy often exhibited over-folded conformations, as seen with MGA and htRNA^Gly-GCC^ (Figure 1C and Figure 3C) [44]. Notably, among the top ten lowest energy structures, we identified MGA structures that were more native-like than the lowest energy structure. This finding aligns with the documented limitations of Rosetta FARFAR2, where lower-energy conformations are favored over known structures, especially in larger RNA molecules (>80 nt) [24,45]. While Rosetta FARFAR2 may perform well with helical structures like pre-miRNAs, the three-residue fragmentation process can disrupt the translation of long-range interactions, potentially explaining its failure in preserving specific topologies and recognizing isoacceptor conservation, as seen between htRNA^Gly-CCC^ and htRNA^Gly-GCC^. On the other hand, its energy-based approach likely facilitates the construction of more compact and organized structures with proper secondary structure inputs, indicating possible refinement. Its high flexibility, sensitivity, and robust sampling capability make Rosetta FARFAR2 an effective means when supplementary experimental data or template structures are available.

AlphaFold 3 directly produces RNA 3D structures from primary sequence inputs, which more closely resemble those in experimental structure databases across all RNAs in the current study while giving variable results and low confidence in predicting 3D structures for RNAs lacking experimental structure. It consistently predicts the inverted “L” shape structure [46] for a tRNA such as the htRNA^Gly-GCC^ and the segment within BioRNA while lacking experimental structures. AlphaFold 3 leverages a multiple sequence alignment (MSA) module in its RNA 3D structure prediction, which utilizes genetic databases such as Rfam, RNACentral, and the Nucleotide Collection to identify homologous sequences and structure templates. AlphaFold 3 then constructs vector–matrix representations of single and paired residues and refines to encode structural details and long-range interactions derived from the MSA. This information package is processed by a diffusion module, a trained deep-learning AI algorithm that operates with atomic coordinates to generate the final 3D structure. Additionally, AlphaFold 3 estimates local prediction confidence through the predicted local distance difference test (pLDDT), generating the ribbon structure that indicates prediction confidence [23]. The MSA-based approach accounts for the overall superior performance by AlphaFold 3 in reproducing structural elements like D/T loop interactions in htRNA^Gly^ isoacceptors. However, for the predicted htRNA^Gly-CCC^ 3D structure, while it remains below the RMSD_100_ threshold, AlphaFold 3 does not fully refine it to match the crystal structure folding, instead maintaining the “L” shaped structure. This suggests that AlphaFold 3 does not necessarily prioritize matching the specific structure template but considers overall structural conservation across all relevant experimental structures.

In addition, AlphaFold 3 allows the inclusion of several common, post-transcriptionally modified nucleotides, such as ψ, 5mU, and Gm, in a primary sequence input for RNA 3D structure prediction, whereas RNAComposer and Rosetta FARFAR2 do not. Specific modifications found in natural RNAs (e.g., tRNAs) and chemo-engineered, therapeutics RNAs (e.g., siRNAs) are well known for their effects on RNA properties and clinical relevance, including metabolic stability, bioactivity, safety, and association with particular diseases [2,4,47,48], which are undoubtedly related to structural changes. While AlphaFold 3 predicted 3D structures are not visually obvious for the modified and unmodified nedosiran siRNA or htRNA^Leu-UAA^ examined in the present study, small differences are still indicated by RMSD values along with local alterations. The acceptance of relevant modifications by AlphaFold 3 for RNA 3D structure prediction and its performance are probably related to the current understanding of frequent post-transcriptional modifications and deep learning from available 3D structures with minor modifications, which unfortunately are largely from RNA molecules synthesized in vitro.

Traditionally, fragment-based methods like RNAComposer and Rosetta FARFAR2 have faced challenges related to fragment selection and data divergence [27]. Larger fragments, like those used by RNAComposer, can effectively capture the global folding of experimental structures, but carry the risk of selecting inaccurate reference templates. Smaller fragments, as in Rosetta FARFAR2, avoid such mismatches but require a vast pool of sampled structures, often resulting in lower-energy conformations that may not reflect the experimental structures. AlphaFold 3, while also working with small fragments of paired residues, enhances its predictions by leveraging evolutionary conservation data to refine the scope of possible conformations. While there is slightly poorer performance in predicting an MGA structure that might be related to the absence or presence of binding ligand TMR, the 3D structures of all other larger RNA molecules predicted by AlphaFold 3 in this study closely align with the experimental structures. This high accuracy is achieved through a trained neural network operating with MD principles to refine and construct the most representative 3D structures. Although this approach may trade some flexibility and sensitivity, AlphaFold 3 has proven to be a robust computational tool with strong convergence and validation through pLDDT for its predictions, potentially overcoming the limitations often associated with fragment-based assembly methods. Nevertheless, this study is limited to a small number of RNA molecules, and research on more and larger RNAs, including ribosomal and messenger RNAs, as well as their interactions with RNA binding proteins and small-molecule modulators critical for general biomedical research and drug screening, are highly warranted.

In conclusion, compared to the easy use of primary sequence input by AlphaFold 3, RNAComposer and Rosetta FARFAR2 require a constrained RNA secondary structure input that largely influences their 3D structure predictions. Overall, AlphaFold 3 outperforms RNAComposer and Rosetta FARFAR2 to computationally offer RNA 3D structures closely characterize experimental results with high confidence while indicating low confidence for RNA molecules or segments lacking experimental structures. Such computational predictions inevitably demand experimental validation, while the needs for experimental characterization of more RNA 3D structures to machine learning or refinement are obvious.

## 4. Materials and Methods

### 4.1. General Workflow

**Figure 7:**
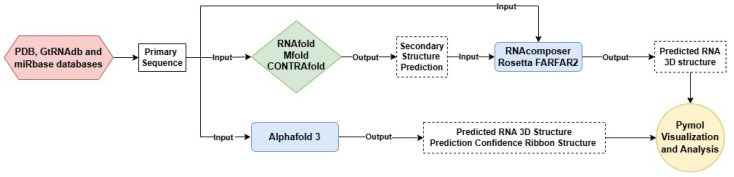
The workflow for computational prediction of RNA 3D structures. It starts by retrieving primary sequences from relevant databases such as PDB, GtRNAdb, and miRBase. The RNA secondary structures are obtained by using RNAfold, Mfold, or CONTRAfold, and then used by conventional tools (RNAComposer and Rosetta FARFAR2) to generate 3D structures. By contrast, the primary sequence is fed directly into AlphaFold 3 for 3D structure prediction. The resultant structures are visualized and analyzed using PyMOL 3.0, facilitating a detailed comparison of specific structures.

Our workflow to predict RNA 3D structures is illustrated in Figure 7. First, we retrieved target RNA primary sequences from relevant publications or databases such as PDB, GtRNAdb, and miRbase. Second, we submitted primary sequences to RNAfold, Mfold, or CONTRAfold for secondary structure prediction. Third, the predicted secondary structures were input into RNAComposer and Rosetta FARFAR2 to obtain RNA 3D structures. On the other hand, the primary sequence was directly input into AlphaFold 3 for 3D structure prediction, which includes built-in confidence analysis. All 3D structures were visualized and analyzed using PyMOL 3.0.

### 4.2. RNA Primary Sequence

The primary sequences of individual RNAs used in this study were acquired from relevant literature and publicly available databases, such as PDB, GtRNAdb, and miRbase. In addition, the assembly of BioRNA uses the pre-miRNA (e.g., hsa-pre-let-7a) to replace the anticodon of tRNA (e.g., htRNA^Gly-GCC^).

### 4.3. RNA Secondary Structure Predictions

In this study, we employed both physics-based and probabilistic models to predict RNA secondary structures. Traditionally, physics-based models, such as those used by RNAfold in the ViennaRNA package, have been dominant for single-sequence RNA secondary structure prediction. These models rely on experimentally derived thermodynamic parameters, such as minimized free energy, to predict the most stable RNA structure. RNAfold, in particular, has been a leading tool in this field due to its foundation in thermodynamic principles and its continuous optimization based on empirical measurements. The current version, ViennaRNA 2.6.3, offers significant improvements in performance over earlier versions [42,49]. On the other hand, probabilistic models like CONTRAfold, which are based on machine learning and statistical training, offer a more flexible approach for modeling complex RNA structures, including multi-branch loops. While CONTRAfold has outperformed earlier versions of RNAfold, we opted to use both tools depending on the RNA molecule under study. RNAfold was employed for molecules with well-characterized experimental structures, ensuring reliable predictions based on well-established thermodynamic principles. However, for RNA molecules without experimentally determined structures, such as htRNA^Gly-GCC^ and BioRNA, we found that CONTRAfold provided higher quality predictions, especially in cases where more complex structural interactions were anticipated. Since AlphaFold 3, another tool we used in this study, does not rely on user-defined secondary structures, we aimed to use the highest quality secondary structure predictions for RNAComposer and FARFAR2. This also allowed us to test the efficacy of these secondary structure predictions in guiding accurate 3D structure prediction.

We used the RNAfold web server with default settings to calculate the optimal secondary structures for our RNA molecules. RNAfold generates the following two predictions: one based on absolute minimum free energy (MFE) and another representing the most likely secondary structure among all possible ones (Centroid) [38]. When the two predictions aligned, this indicated high confidence in the predicted structure (Figure S1A,B,D). Similarly, we employed the CONTRAfold web server with default settings. CONTRAfold takes a single RNA sequence in FASTA format and outputs a secondary structure prediction in dot–bracket format. The resulting structures were visualized using the forna tool, as CONTRAfold does not provide built-in visualization [42].

The RNAfold server is accessible at

http://rna.tbi.univie.ac.at//cgi-bin/RNAWebSuite/RNAfold.cgi.

The CONTRAfold server is accessible at

http://contra.stanford.edu/contrafold/server.html.

The forna is accessible at

http://rna.tbi.univie.ac.at/forna/#:~:text=forna%20is%20a%20RNA%20secondary%20structure%20visualization%20tool%20which%20is.

### 4.4. Computational 3D Structure Prediction

#### 4.4.1. RNAComposer

RNAComposer, accessible via an online web server, was used for fully automated 3D RNA structure prediction in this study. For each RNA sequence, complemented by a user-defined secondary structure input, a single 3D structure was generated using the default interactive mode and is presented in this study [28].

The RNAComposer is accessible at https://rnacomposer.cs.put.poznan.pl/.

#### 4.4.2. Rosetta FARFAR2

The Rosetta FARFAR2 protocol was performed using the ROSIE web server. The inputs included the RNA sequence and its predicted secondary structure, processed with default parameters. The protocol was configured to generate a maximum of 2000 structures without constraints or template files. From the output, the top ten lowest energy cluster centers were identified and downloaded. The lowest energy cluster center was selected for presentation in this study [44].

The ROSIE FARFAR2 is accessible at https://rosie.rosettacommons.org/farfar2.

#### 4.4.3. AlphaFold 3

The RNA 3D structures were predicted using the AlphaFold server by inputting the primary RNA sequences. The server generated five structures in its default mode. Among these, the structure with the highest prediction confidence, labeled “_model_0,” was selected for presentation. The confidence ribbon structure, as displayed by the web server, was captured and included in this study [23].

The AlphaFold Server is accessible at https://alphafoldserver.com/.

### 4.5. 3D Structure Visualization and Validation

The predicted 3D structures from the computational tools were annotated and analyzed using the PyMOL molecular graphics system version 3.0. Visualization of the individual RNA 3D models was performed under maximum quality settings in PyMOL 3.0. The images were rendered with ray-traced transparent backgrounds for a clean presentation. Models were adjusted to optimal viewing angles and orientations to enhance clarity and ensure accurate representation of the RNA structures. For quantitative structural comparisons, the ‘align’ command was employed with the ‘cycles = 0′ parameter to ensure a comprehensive comparison of the models. The RMSD values were calculated and displayed in the terminal dialogue, providing a metric for assessing the structural alignment between models [50].

The PyMOL 3.0 is accessible at https://pymol.org/.

## Data Availability

No datasets were generated during the study. The raw data supporting the conclusions of this article will be made available by the authors upon request.

## Supplementary Materials

The following supporting information can be downloaded at: https://www.mdpi.com/article/10.3390/ncrna10060055/s1, Table S1: Primary sequences of RNAs examined in this study and their secondary structures predicted by RNAfold and CONTRAfold. Table S2: Primary sequences of nedosiran siRNA and htRNA^Leu-UAA^ with and without modifications. Figure S1: The secondary structures of (A) MGA, (B) htRNA^Gly-CCC^, (C) htRNA^Gly-GCC^, (D) hsa-pre-let-7a, and (E) BioRNA^Gly^/pre-let-7a predicted by RNAfold (left) and CONTRAfold (right). Figure S2: The 3D structures of htRNA^Gly-CCC^ predicted by RNAComposer (B) and Rosetta FARFAR2 (C) when its secondary structure predicted by CONTRAfold was used. Figure S3: The 3D structures of htRNA^Gly-GCC^ predicted by RNAComposer (A) and Rosetta FARFAR2 (B) failed to recapitulate the inverted “L” shape of a typical tRNA structure when its secondary structure predicted by RNAfold was used. Figure S4: The 3D structures of human pre-let-7a generated by RNAComposer (A) and Rosetta FARFAR2 (B) from the secondary structure predicted by CONTRAfold. Figure S5: The 3D structures of BioRNA^Gly^/pre-let-7a predicted by RNAComposer (A) and Rosetta FARFAR2 (B) when its secondary structure predicted by RNAfold was used.
