# Supplementary material for: Comparison of Three Computational Tools for the Prediction of RNA Tertiary Structures
Source: Noncoding RNA. 2024 Nov 8;10(6):55. doi: 10.3390/ncrna10060055 (PMC11587127; doi:10.3390/ncrna10060055)

Supplementary Data

Comparison of Three Computational Tools for the Prediction of RNA Tertiary Structures

Frank Y. Mao, Mei-Juan Tu, Gavin M. Traber and Ai-Ming Yu*

Department of Biochemistry and Molecular Medicine, University of California- Davis, School of Medicine, Sacramento, CA 95817, USA

Supplemental Tables S1 and S2

Supplemental Figures S1 and S5

Table S1. Primary sequences of RNAs examined in this study and their secondary structures predicted by RNAfold and CONTRAfold. Anticodons are marked in red.

RNAs	Program	Primary sequence	Secondary structure predicted
MGA	RNAfold	GGAUCCCGACUGGCGAGAGCCAGGUAACGAAUGGAUCC	((((((((.(((((....)))))....))...))))))
	CONTRAfold		((((((((.(((((....)))))....))...))))))
htRNA$^{Gly-CCC}$	RNAfold	GCGCCGCUGGUGUAGUGGUAUCAUGCAAGAUU**CCC**AUUCUUGCGACC<br>CGGGUUCGAUUCCCGGCGGCGCACCA	((((((((((((......))))).((((((.......))))))...(((((.......))))))))))))....
	CONTRAfold		(((((((..(((.........)))((((((.......))))))...(((((.......))))))))))))....
htRNA$^{Gly-GCC}$	RNAfold	GCAUGGGUGGUUCAGUGGUAGAAUUCUCGCCU**GCC**ACGCGGGAGGCC<br>CGGGUUCGAUUCCCGGCCCAUGCA	(((((((.(((((.......)))))...((((.((....)).))))(((((.......)))))))))))).
	CONTRAfold		(((((((..((((.......))))((((((.......))))))...(((((.......)))))))))))).
hsa-pre-let-7a	RNAfold	UGGGAUGAGGUAGUAGGUUGUAUAGUUUUAGGGUCACACCCACCACU<br>GGGAGAUAACUAUACAAUCUACUGUCUUUCCUA	(((((.(((((((((((((((((((((.....(((...((((....)))).)))))))))))))))))))))))))))))
	CONTRAfold		(((((.(((((((((((((((((((((...........(((......)))....))))))))))))))))))))))))))
htRNA$^{Gly-GCC}$/hsa-pre-let-7a	RNAfold	GCAUGGGUGGUUCAGUGGUAGAAUUCUCGCCUUGGGAUGAGGUAGUA<br>GGUUGUAUAGUUUUAGGGUCACACCCACCACUGGGAGAUAACUAUAC<br>AAUCUACUGUCUUUCCUAACGCGGGAGGCCCGGGUUCGAUUCCCGGC<br>CCAUGCA	(((((((....((.......))..((((((.((((((.(((((((((((((((((((((.....(((...((((....)))).)))))))))<br>))))))))))))))))))))).))))))....((((.......)))).))))))).
	CONTRAfold		(((((((..((((.......))))((((((.(((((..(((((((((((((((((((((...........(((......)))....)))))))<br>)))))))))))))).))))).))))))...(((((.......)))))))))))).

Table S2. Primary sequences of nedosiran siRNA and htRNA$^{\text{Leu-UAA}}$ with and without modifications. Note that AlphaFold 3 only accepts part of these modifications. All modified bases are underlined. A, adenosine; AdemA, GalNAc aminosugar-conjugated adenosine; AdemG, GalNAc aminosugar-conjugated guanosine; Af, 2′-fluoroadenosine; Am, 2′-O-methyladenosine; C, cytidine; Cf, 2′-fluorocytidine; Cm, 2′-*O*-methylcytidine; G, guanosine; Gf, 2′-fluoroguanosine; Gm, 2′-*O*-methylguanosine; m1G, *N*1-methylguanosine; s, phosphorothioate; U, uridine; Uf, 2′-fluorouridine; Um, 2′-*O*-methyluridine; Um*, 2′-*O*-methyl-4′-*O*-((methoxy)phosphoryl)methyluridine; ψ, pseudourdine; m5U, 5-methyluridine; D, dihydrouridine.

**RNA**	**Primary Sequences without and with specific modifications**	
Nedosiran (Rivfloza)	Modified (Nedosiran)	Sense: 5’-AmsUmGfUmUfGmUmCfCfUfUfUmUfUmAfUmCfUmGmAmGmCmAmGmCm-Cm-AdemG-AdemA-AdemA-AdemA-GmGmCmUmGmCm-3’<br>Antisense: 3’-GmsGmsUmAmCfAmAfCmAfGmGmAmAfAfAmAfGmAfGfsAfsCfsUm*-5’
	Modifications taken by AlphaFold 3	Sense: 5’ –AmUmGUmUGmUmCCUUUmUUmAUmCUmGmAmGmCmAmGmCmCmAAAAGmGmCmUmGmCm-3’<br>Antisense: 3’-GmGmUmAmCAmACmAGmGmAmAAAmAGmAGACUm-5’
	Unmodified	Sense: 5’-AUGUUGUCCUUUUUAUCUGAGCAGCCAAAAGGCUGC-3’<br>Antisense: 3’-GGUACAACAGGAAAAAGAGACU-5’
Human Leucyl-tRNA (htRNA$^{\text{Leu-UAA}}$)	Modified	5’-ACCAGGAUGGCCGAGDGmGDDAAGGCGUUGGACU**UAA**GAUCCAAUGGACAUAUGUCCGCGUGGG m5UψCGAACCCCACUCCUGGUACCA-3’
	Modifications taken by AlphaFold 3	5’-ACCAGGAUGGCCGAGUGmGUUAAGGCGUUGGACU**UAA**GAUCCAAUGGACAUAUGUCCGCGUGGG m5UψCGAACCCCACUCCUGGUACCA-3’
	Unmodified	5’-ACCAGGAUGGCCGAGUGGUUAAGGCGUUGGACU**UAA**GAUCCAAUGGACAUAUGUCCGCGUGGG UUCGAACCCCACUCCUGGUACCA-3’

Figure S1. The secondary structures of (A) MGA, (B) htRNA$^{Gly\text{-}CCC}$, (C) htRNA$^{Gly\text{-}GCC}$, (D) hsa-pre-let-7a, and (E) BioRNA^{Gly}/pre-let-7a predicted by RNAfold (left) and CONTRAfold (right) that were used as inputs for 3D structure prediction by RNAComposer and Rosetta FARFAR2.

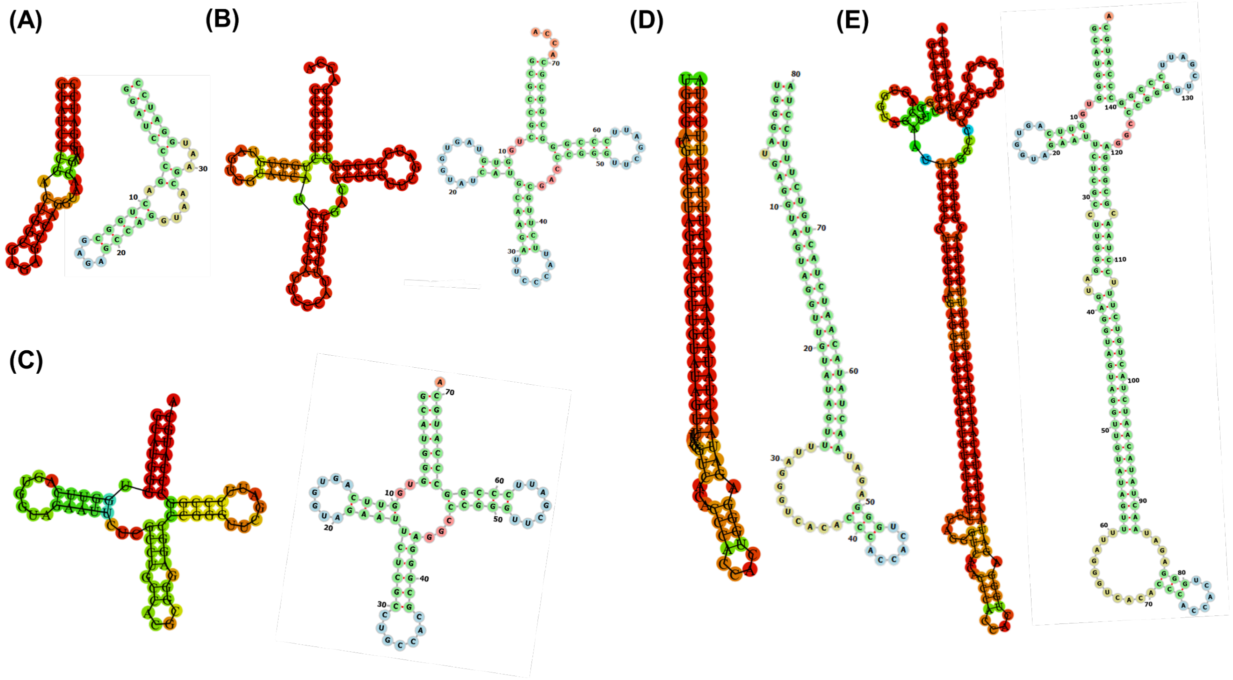

Figure S2. The 3D structures of human glycyl-tRNA-CCC (htRNA$^{Gly\text{-}CCC}$) predicted by RNAComposer (B) and Rosetta FARFAR2 (C) when its secondary structure predicted by CONTRAfold (Table S1 and Figure S1) was used, which showing an RMSD of 5.899 Å and 12.734 Å, respectively, when compared to the crystal structure (A) (PDB ID: 5E6M). Rosetta FAFAR2 now failed to recapitulate the inverted "L" shape of a typical tRNA structure, whereas RNAComposer did. Acceptor arm (wheat tint), D arm (cyan), T arm (yellow), and anticodon arm (orange).

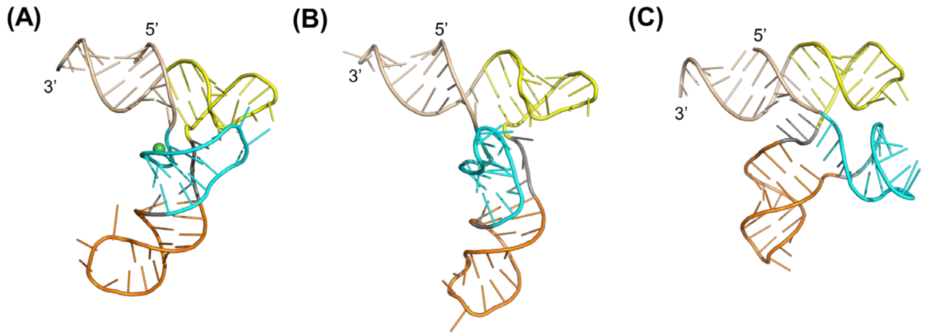

Figure S3. The 3D structures of human glycyl-tRNA-GCC (htRNA$^{Gly-GCC}$) predicted by RNAComposer (**A**) and Rosetta FARFAR2 (**B**) failed to recapitulate the inverted "L" shape of a typical tRNA structure when its secondary structure predicted by RNAfold (Table S1 and Figure S1) was used. Acceptor arm (wheat tint), D arm (cyan), T arm (yellow), and anticodon arm (orange).

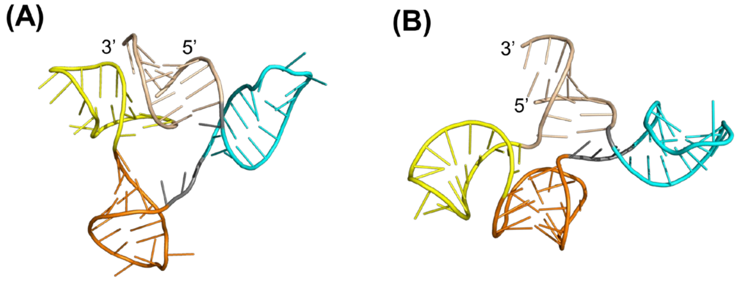

Figure S4. The 3D structures of human precursor microRNA let-7a-1 (pre-let-7a) generated by RNAComposer (**A**) and Rosetta FARFAR2 (**B**) from the secondary structure predicted by CONTRAfold (Table S1 and Figure S1). When compared to the cryo-EM structure (**A**) (PDB ID: 5ZAL), an RMSD of 10.285 Å (**B**) and 10.015 Å (**C**) was noted, respectively. RNAComposer predicted a structure with a clear internal loop (**B**), while Rosetta FARFAR2 predicted a compact structure with a bulge loop of less unpaired bases (**C**). Rather, both exhibit a sharp turn to offer an "L" sharp structure. The pre-let-7a segment: let-7a-1-5p (red), let-7a-1-3p (green), distal loop (pink; orange in ribbon form).

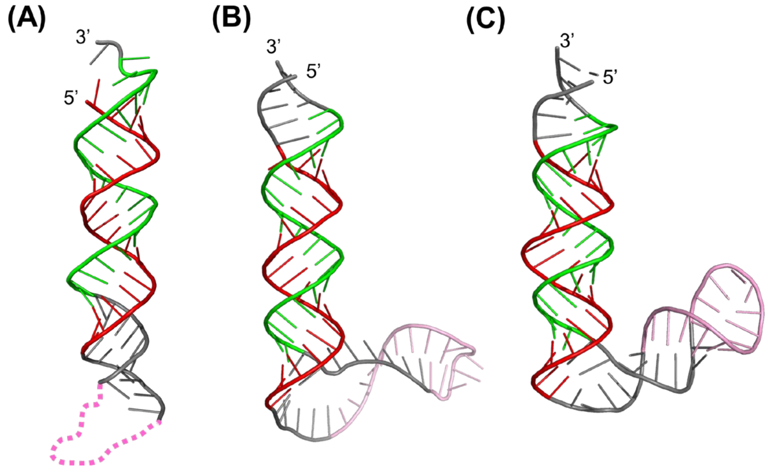

Figure S5. The 3D structures of human tRNA$^{Gly-GCC}$-fused hsa-pre-let-7a (BioRNA^{Gly}/pre-let-7a) predicted by RNAComposer (**A**) and Rosetta FARFAR2 (**B**) when its secondary structure predicted by RNAfold (Table S1 and Figure S1) was used. Acceptor arm (wheat tint), D arm (cyan), T arm (yellow), and anticodon arm (orange); the pre-let-7a segment: let-7a-1-5p (red), let-7a-1-3p (green), distal loop (pink; orange in ribbon form).

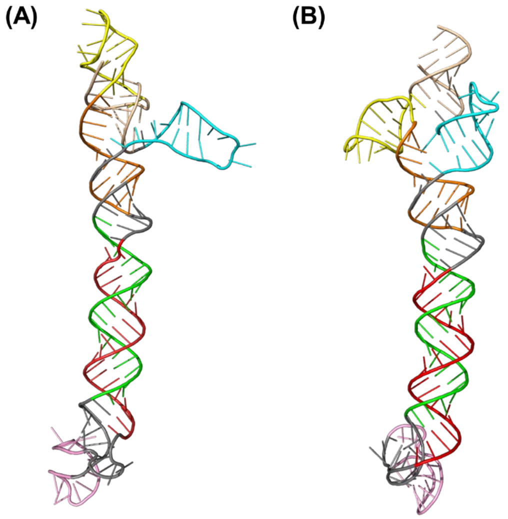

Supplement: Supplementary file 1 [file ncrna-10-00055-s001.zip › ncrna-3267771-supplementary.pdf]
